# Supplementary material for: The Impact of Serum Protein Adsorption on PEGylated NT3–BDNF Nanoparticles—Distribution, Protein Release, and Cytotoxicity in a Human Retinal Pigmented Epithelial Cell Model
Source: Pharmaceutics. 2023 Aug 30;15(9):2236. doi: 10.3390/pharmaceutics15092236 (PMC10537189; doi:10.3390/pharmaceutics15092236)
Supplement: Supplementary file 1 [file pharmaceutics-15-02236-s001.zip › pharmaceutics-2530635-supplementary.pdf]

# Supplementary Materials

# The Impact of Serum Protein Adsorption on PEGylated NT3–BDNF Nanoparticles—Distribution, Protein Release, and Cytotoxicity in a Human Retinal Pigmented Epithelial Cell Model

Maria Dąbkowska [1,*,†], Alicja Kosiorowska [1,2,†] and Bogusław Machaliński [2]

[1] Independent Laboratory of Pharmacokinetic and Clinical Pharmacy, Rybacka 1, 70-204 Szczecin, Poland; alicjakosiorowska@gmail.com

[2] Department of General Pathology, Pomeranian Medical University, Rybacka 1, 70-204 Szczecin, Poland; boguslaw.machalinski@pum.edu.pl

[*] Correspondence: maria.dabkowska@pum.edu.pl

[†] These authors contributed equally to this work.

**Determining the size and nanoparticle concentration.**

## Part 1

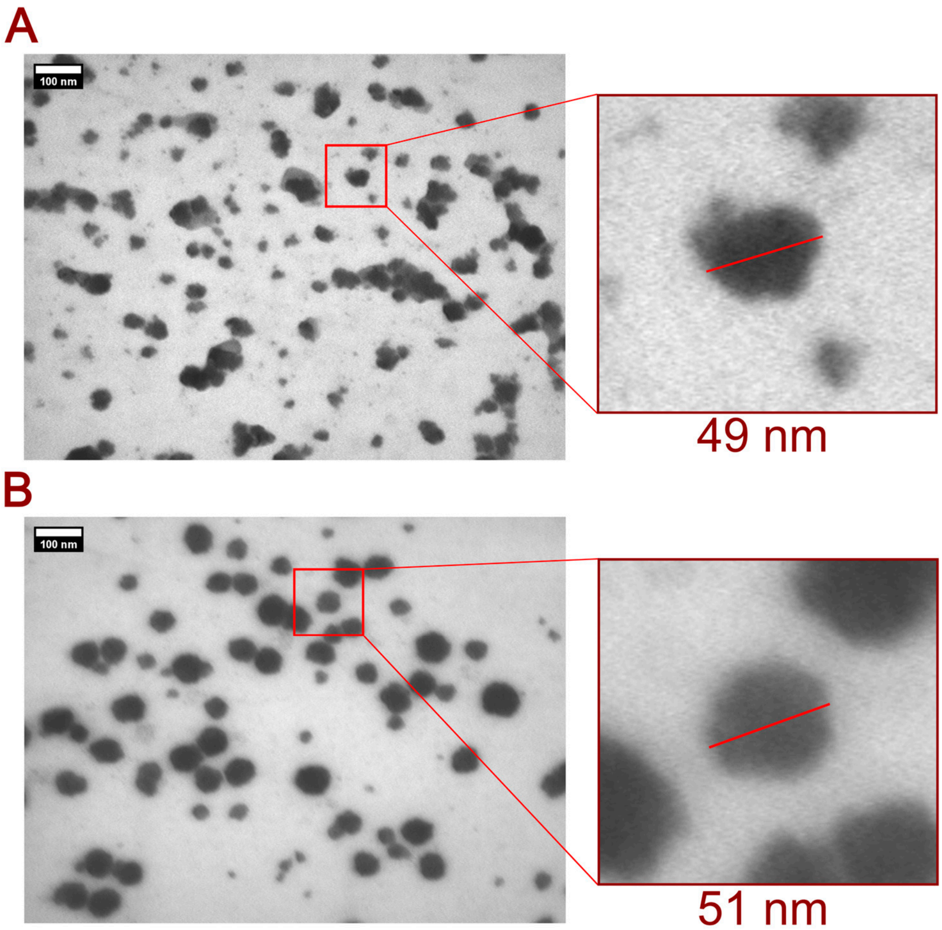

# Part 2

## A)

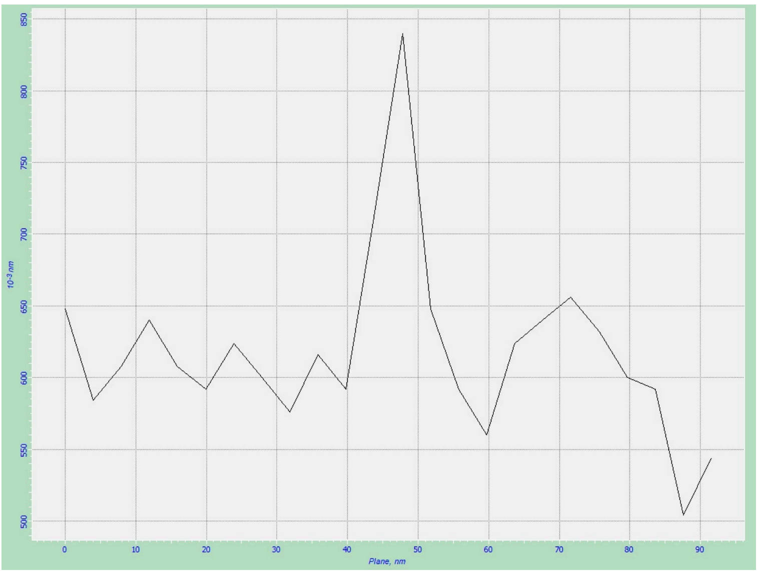

850
800
750
700
650
600
550
500
10-3 nm
0
10
20
30
40
50
60
70
80
90
Plane, nm


## B)

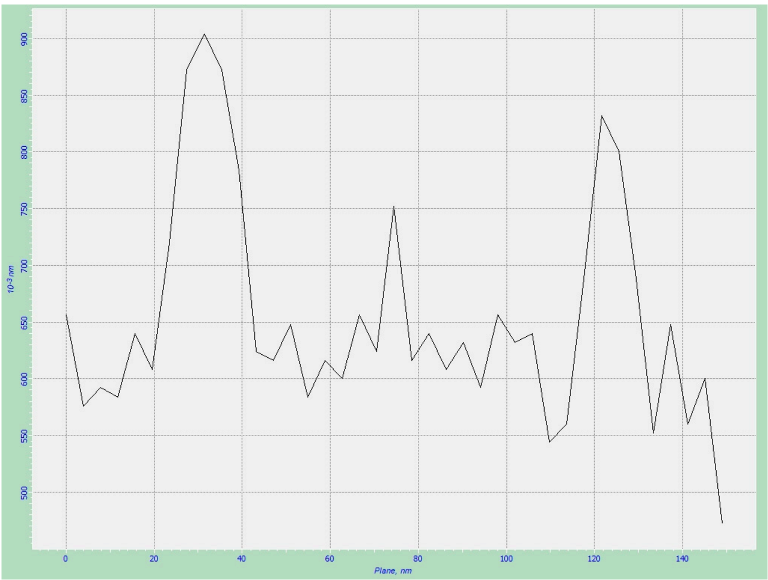

900
850
800
750
700
650
600
550
500
10-3 nm
0
20
40
60
80
100
120
140
Plane, nm

**C)**

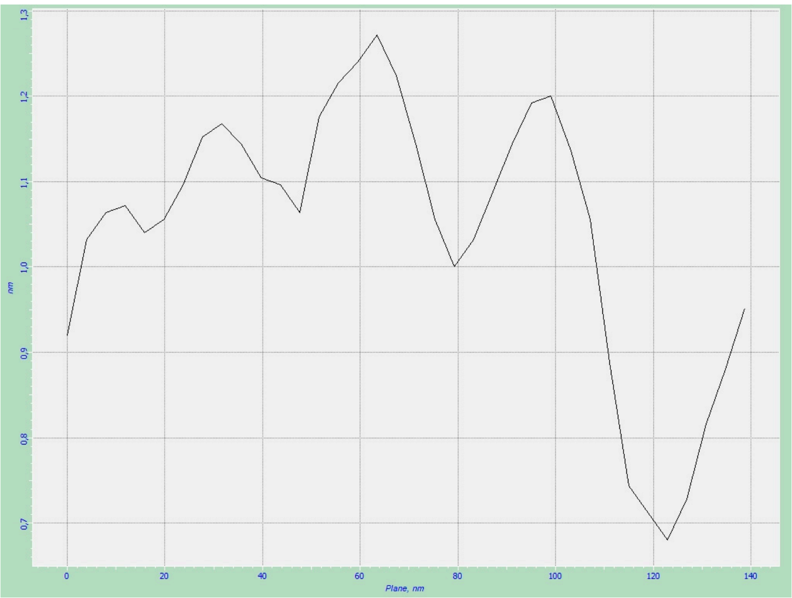


**Figure S1.** (Part 1) SEM images of PEGylated NT3-BDNF nanoparticles deposited from 0.15M, pH 7.4: PBS, containing various concentration of recombinant neurotrophins: 1 $mgL^{-1}$ NT3 and 1 $mgL^{-1}$ BDNF (Figure S1.1.A), 5 $mgL^{-1}$ NT3 and 5 $mgL^{-1}$ BDNF (Figure S1.1.B). The images on the left and right side corresponds to a 100 000 magnification. Scale bar is 100 nm. (Part 2) AFM images of PEGylated NT3-BDNF nanoparticles deposited onto the mica surface at 0.15M, pH 7.4 in PBS for various concentrations of recombinant neurotrophins. The structures of PEGylated NT3-BDNF nanoparticles after cross-section: 0.1 $mgL^{-1}$ (part A), 1$mgL^{-1}$ (part B), 5$mgL^{-1}$ (part C).

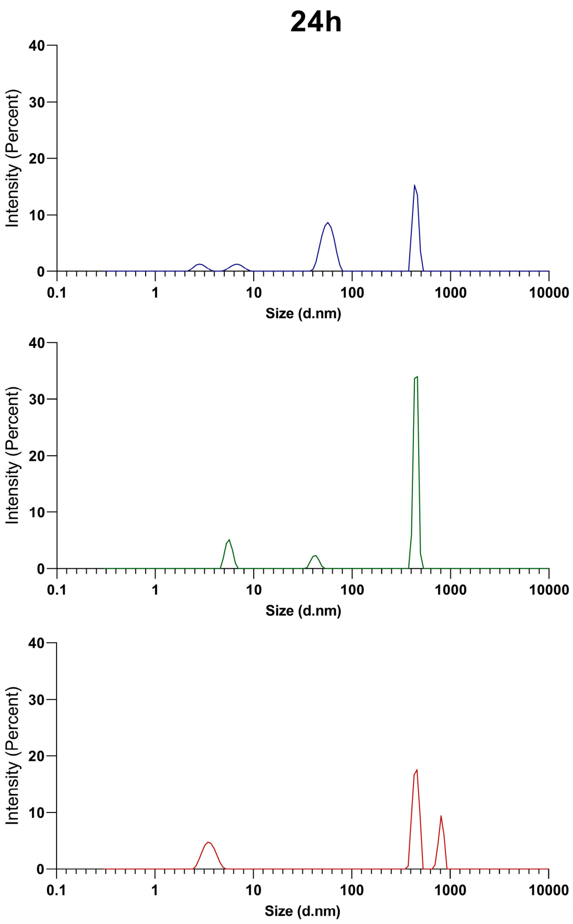


**Figure S2**. Size distribution by MADLS, of PEGylated NT3-BDNF nanoparticles with different neurotrophin concentrations: 0.1 $mgL^{-1}$ (blue curve), 1$mgL^{-1}$ (green curve), 5$mgL^{-1}$ (red curve) over 24h after formulation. The intensity (percent) indicates the sizes of the frequency of the most common particles.

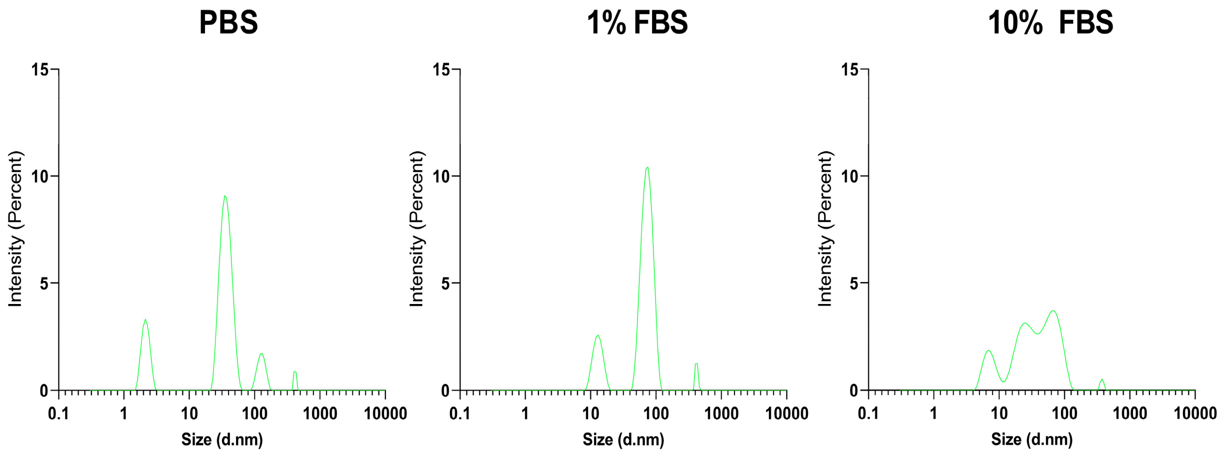


**Figure S3.** Dependence of NPs aggregation on various bulk conditions (PBS, PBS supplemented with 1% FBS, and PBS supplemented with 10% FBS) was shown at 72 hours. PEGylated NT3-BDNF nanoparticles aggregation triggered by FBS supplementation showed variable dynamics depending on neurotrophins concentration: 1mgL$^{-1}$ (green curve).

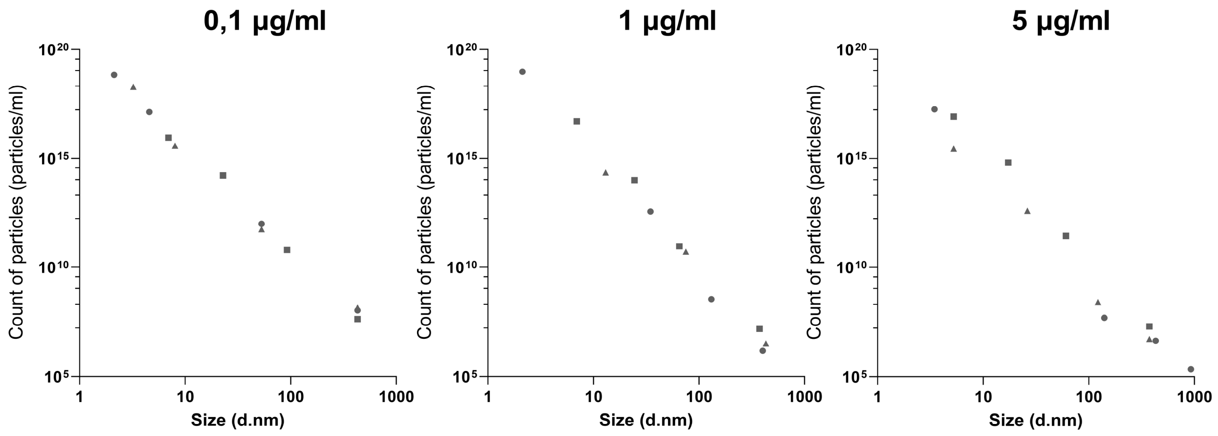


**Figure S4.** Dependences of PEGylated BDNF-NT3 nanoparticles concentration on various bulk conditions: PBS (circle), PBS supplemented with 1% FBS (triangle), and PBS supplemented with 10% FBS (square) were shown at 72 hours cells treatment. The concentration of nanoparticles is expressed in the unit [the number of particles/ml].

| NPs concentration [particle ml-1] / PEGylated NT3-BDNF NPs [mgL-1] | 2h | | | | 24h | | | 72h | | | |
|---|---|---|---|---|---|---|---|---|---|---|---|
| | Peak 1 | Peak 2 | Peak 3 | Peak 4 | Peak 1 | Peak 2 | Peak 3 | Peak 1 | Peak 2 | Peak 3 | Peak 4 |
| | x $10^{16}$ | x$10^{9}$ | x$10^{6}$ | x$10^{5}$ | x$10^{17}$ | x$10^{10}$ | x$10^{7}$ | x$10^{19}$ | x$10^{18}$ | x$10^{11}$ | x$10^{8}$ |
| **0.1** | 6.31 | 4.52 | 8.66 | - | 3.32 | 7.49 | 2.08 | 7.04 | 6.9 | 9.96 | 1.08 |
| **1** | 8.45 | 0.009 | 10.5 | - | 0.3 | 0.68 | 6.38 | 0.96 | - | 0.004 | 0.02 |
| **5** | 18.3 | 0.05 | 4.39 | 2.17 | 1.48 | 0.001 | 0.6 | 0.04 | - | 0.001 | 0.01 |

**Table S1.** The correlations between concentration of PEGylated BDNF-NT3 nanoparticles as a function of various neurotrophin concentrations, bulk conditions: PBS, and time points.

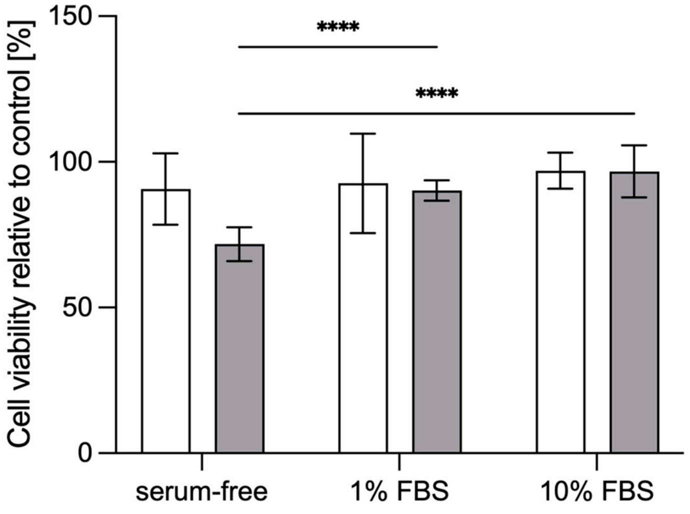


**Figure S5. ARPE-19 cell viability.** Effect of serum concentration (serum-free, 1% FBS and 10% FBS) and PEGylated nanoparticles NT3-BDNF on ARPE-19 cell viability over 72 hours as measured with alamarBlue® assay. ARPE-19 cell line was exposed for up to 72 hours to increasing concentrations of BDNF-NT3 released from PEGylated nanoparticles at 1mgL$^{-1}$ (n=12); Data was analysed using repeated measures ANOVA with Tukey's post-test; ****p <0.0001.

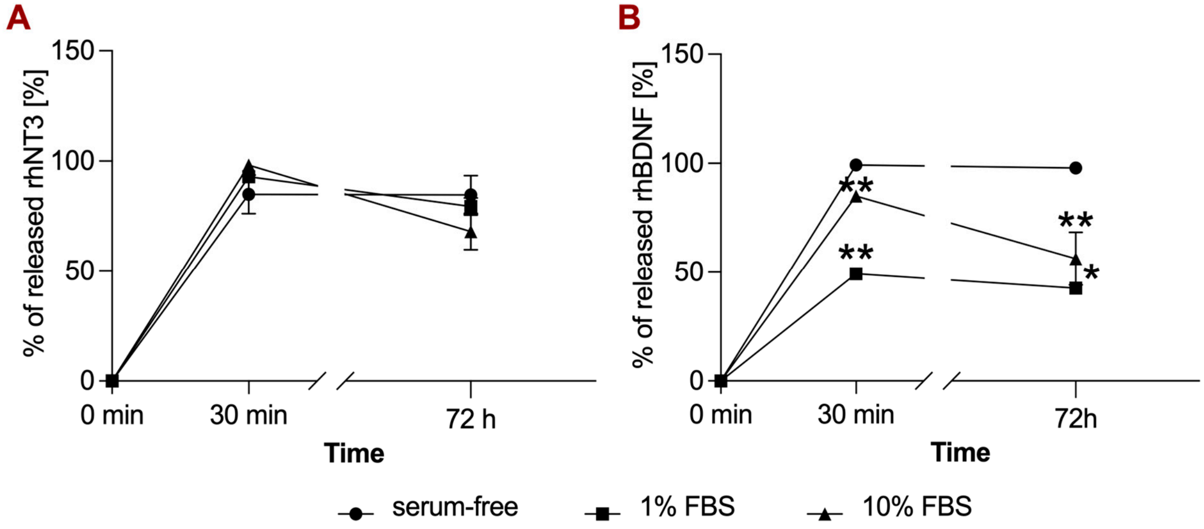


**Figure S6. Release of rhNT3 (*A*) and rhBDNF (*B*) from PEGylated NT3-BDNF nanoparticles *in a cell free system.***

PEGylated NT3-BDNF nanoparticles (1 $mgL^{-1}$) were incubated in serum-free, 1% and 10% FBS for up to 72 hours with constant mixing. After each time point a sample was removed and released rhBDNF and NT3 were separated by ultracentrifugation. Released levels were measured with ELISA. Data was analysed using repeated measures ANOVA with Tukey's post-test comparing protein release profile in serum-free DMEM F-12 and DMEM F-12 + 1% FBS, *p < 0.05, **p < 0.005.

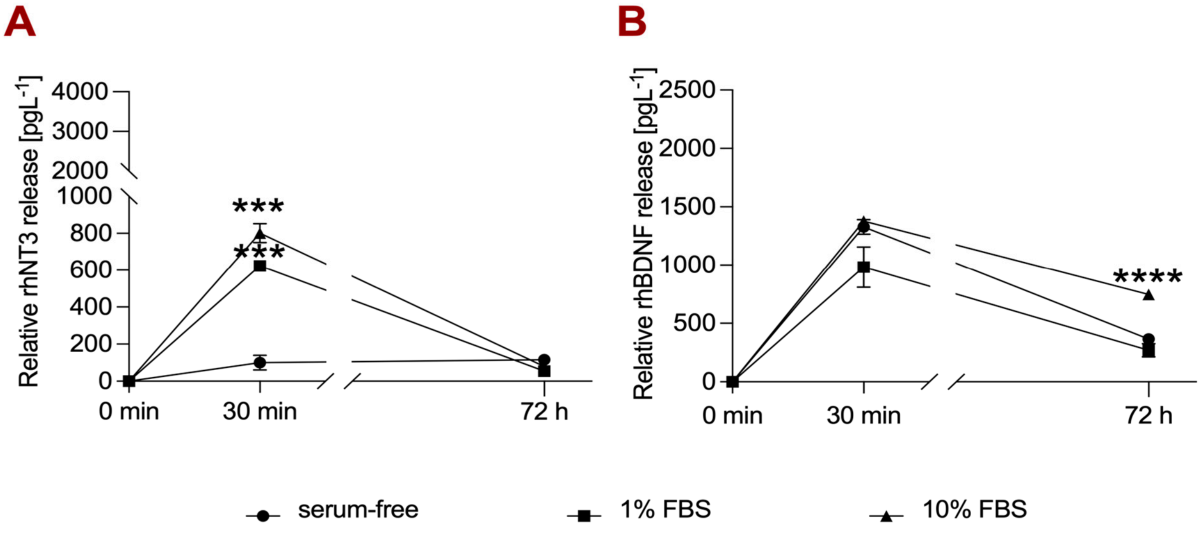


**Figure S7. PEGylated NT3-BDNF NPs release profile in vitro**.

NT3-BDNF PEGylated NPs (1 $mgL^{-1}$) show time-dependent release over 72h period for rhNT3 (a) and rhBDNF (b). Results are mean ± SD of three replicates. Data was analysed using repeated measures ANOVA with Tukey's post-test comparing protein release profile in serum-free DMEM F-12 and DMEM F-12 + 1% FBS, ***p < 0.005, ****p < 0.001.

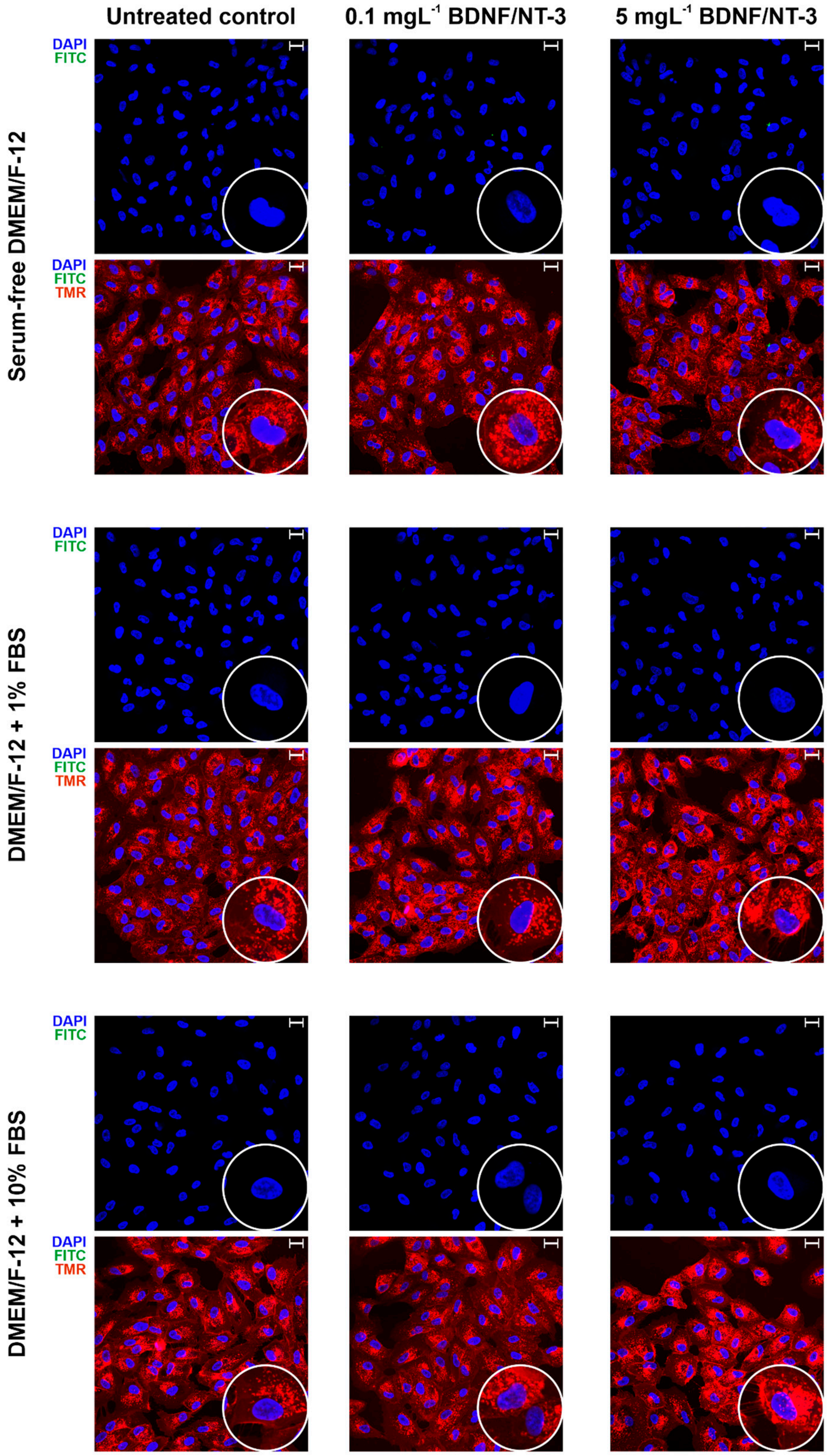


**Figure S8. Cellular internalisation of PEG-ylated BDNF/NT-3 nanoparticles after 30 minutes.**
The ARPE-19 cells cultured in different DMEM/F-12 media conditions: serum-free, complemented with 1% of FBS and 10% FBS, were incubated for 30 minutes with PEGylated NT3-BDNF NPs (0.1 $mgL^{-1}$ or 5 $mgL^{-1}$). Green fluorescence (FITC) represents nanoparticles, blue (DAPI) – cell nuclei, red (tetramethylrhodamine) – surface glycoproteins. The scale bar is 20 µm. All images include inserts with 3x magnification.
